# Supplementary material for: Prevalence of chronic obstructive pulmonary disease at high altitude: a systematic review and meta-analysis
Source: PeerJ. 2020 Apr 3;8:e8586. doi: 10.7717/peerj.8586 (PMC7134014; doi:10.7717/peerj.8586)
Supplement: Supplemental Information 1 [file peerj-08-8586-s001.docx]

**Appendix S1. Search strategies in databases**

**Search strategy in PubMed**

((((("Altitude"[Mesh]) OR altitudes[Title/Abstract])) OR ((plateau[Title/Abstract]) OR country-level[Title/Abstract]))) AND ((((((("Pulmonary Disease, Chronic Obstructive"[Mesh]) OR (((((((((COPD[Title/Abstract]) OR Chronic Obstructive Pulmonary Disease[Title/Abstract]) OR COAD[Title/Abstract]) OR Chronic Obstructive Airway Disease[Title/Abstract]) OR Chronic Obstructive Lung Disease[Title/Abstract]) OR Airflow Obstruction, Chronic[Title/Abstract]) OR Airflow Obstructions, Chronic[Title/Abstract]) OR Chronic Airflow Obstructions[Title/Abstract]) OR Chronic Airflow Obstruction[Title/Abstract])) OR "Lung Diseases, Obstructive"[Mesh]) OR (((((((Lung Disease, Obstructive[Title/Abstract]) OR Obstructive Lung Disease[Title/Abstract]) OR Obstructive Lung Diseases[Title/Abstract]) OR Obstructive Pulmonary Diseases[Title/Abstract]) OR Obstructive Pulmonary Disease[Title/Abstract]) OR Pulmonary Disease, Obstructive[Title/Abstract]) OR Pulmonary Diseases, Obstructive[Title/Abstract])) OR ((Bronchitis, Chronic[Title/Abstract]) OR Chronic Bronchitis[Title/Abstract])) OR "Pulmonary Emphysema"[Mesh]) OR (((((((((((((((((((((((Emphysemas, Pulmonary[Title/Abstract]) OR Pulmonary Emphysemas[Title/Abstract]) OR Emphysema, Pulmonary[Title/Abstract]) OR Focal Emphysema[Title/Abstract]) OR Emphysema, Focal[Title/Abstract]) OR Emphysemas, Focal[Title/Abstract]) OR Focal Emphysemas[Title/Abstract]) OR Panacinar Emphysema[Title/Abstract]) OR Emphysema, Panacinar[Title/Abstract]) OR Emphysemas, Panacinar[Title/Abstract]) OR Panacinar Emphysemas[Title/Abstract]) OR Panlobular Emphysema[Title/Abstract]) OR Emphysema, Panlobular[Title/Abstract]) OR Emphysemas, Panlobular[Title/Abstract]) OR Panlobular Emphysemas[Title/Abstract]) OR Centriacinar Emphysema[Title/Abstract]) OR Centriacinar Emphysemas[Title/Abstract]) OR Emphysema, Centriacinar[Title/Abstract]) OR Emphysemas, Centriacinar[Title/Abstract]) OR Centrilobular Emphysema[Title/Abstract]) OR Centrilobular Emphysemas[Title/Abstract]) OR Emphysema, Centrilobular[Title/Abstract]) OR Emphysemas, Centrilobular[Title/Abstract]))

**Search strategy in Embase**

Session Results

.......................................................

No. Query Results

#14. #10 AND #13

#13. #11 OR #12

#12. 'high altitude':ab,ti OR 'plateau':ab,ti OR 'country-level':ab,ti

#11. 'altitude'/exp

#10. #5 OR #7 OR #9

#9. #3 OR #8

#8. 'bronchitis chronica':ab,ti OR 'bronchitis, chronic':ab,ti OR 'chronic bronchus

infection':ab,ti

#7. #2 OR #6

#6. 'bulbous emphysema':ab,ti OR 'bullous emphysema':ab,ti OR 'centrilobular emphysema':ab,ti OR 'chronic lung emphysema':ab,ti OR 'emphysema pulmonale':ab,ti OR 'emphysema, bullous':ab,ti OR 'intrapulmonary interstitial emphysema':ab,ti OR 'lobular emphysema':ab,ti OR 'lung bullous emphysema':ab,ti OR 'lung interstitial emphysema':ab,ti OR 'panacinar emphysema':ab,ti OR 'pneumatosis pulmonum':ab,ti OR 'pneumonectasia':ab,ti OR 'pulmonary emphysema':ab,ti OR 'unilateral pulmonary emphysema':ab,ti OR 'volumen pulmonum auctum':ab,ti

#5. #1 OR #4

#4. 'chronic airflow obstruction':ab,ti OR 'chronic airway obstruction':ab,ti OR 'chronic obstructive bronchitis':ab,ti OR 'chronic obstructive bronchopulmonary disease':ab,ti OR 'chronic obstructive lung disorder':ab,ti OR 'chronic obstructive pulmonary disease':ab,ti OR 'chronic obstructive pulmonary disorder':ab,ti OR 'chronic obstructive respiratory disease':ab,ti OR 'copd':ab,ti OR 'lung chronic obstructive disease':ab,ti OR 'lung disease, chronic obstructive':ab,ti OR 'lung diseases, obstructive':ab,ti OR 'obstructive lung disease':ab,ti OR 'obstructive lung disease, chronic':ab,ti OR 'obstructive pulmonary disease':ab,ti OR 'obstructive respiratory disease':ab,ti OR 'obstructive respiratory tract disease':ab,ti OR 'pulmonary disease, chronic obstructive':ab,ti OR 'pulmonary disorder, chronic obstructive':ab,ti

#3. 'chronic bronchitis'/exp

#2. 'lung emphysema'/exp

#1. 'chronic obstructive lung disease'/exp

.......................................................

**Search strategy in Cochrane Library**

ID Search

#1 MeSH descriptor: [Lung Diseases, Obstructive] explode all trees

#2 Pulmonary Disease, Obstructive

#3 Obstructive Pulmonary Diseases

#4 Obstructive Lung Diseases

#5 Obstructive Lung Disease

#6 Lung Disease, Obstructive

#7 Pulmonary Diseases, Obstructive

#8 Obstructive Pulmonary Disease

#9 #1 or #2 or #3 or #4 or #5 or #6 or #7 or #8

#10 MeSH descriptor: [Bronchitis, Chronic] explode all trees

#11 Chronic Bronchitis

#12 #10 or #11

#13 MeSH descriptor: [Pulmonary Emphysema] explode all trees

#14 Emphysema, Centrilobular

#15 Centrilobular Emphysemas

#16 Centrilobular Emphysema

#17 Centriacinar Emphysema

#18 Emphysema, Centriacinar

#19 Emphysemas, Centriacinar

#20 Centriacinar Emphysemas

#21 Emphysemas, Centrilobular

#22 Emphysemas, Pulmonary

#23 Emphysema, Pulmonary

#24 Pulmonary Emphysemas

#25 Panlobular Emphysemas

#26 Panacinar Emphysema

#27 Emphysemas, Panacinar

#28 Emphysema, Panlobular

#29 Panlobular Emphysema

#30 Panacinar Emphysemas

#31 Emphysemas, Panlobular

#32 Emphysema, Panacinar

#33 Emphysemas, Focal

#34 Focal Emphysema

#35 Focal Emphysemas

#36 Emphysema, Focal

#37 #13 or #14 or #15 or #16 or #17 or #18 or #19 or #20 or #21 or #22 or #23 or #24 or #25 or #26 or 27 or #28 or #29 or #30 or #31 or #32 or #33 or #34 or #35 or #36

#38 MeSH descriptor: [Pulmonary Disease, Chronic Obstructive] explode all trees

#39 Chronic Obstructive Lung Disease

#40 Chronic Obstructive Airway Disease

#41 COPD

#42 Chronic Obstructive Pulmonary Disease

#43 COAD

#44 Airflow Obstructions, Chronic

#45 Airflow Obstruction, Chronic

#46 Chronic Airflow Obstruction

#47 Chronic Airflow Obstructions

#48 #38 or #39 or #40 or #41 or #42 or #43 or #44 or #45 or #46 or #47

#49 #9 or #12 or #37 or #48

#50 MeSH descriptor: [Altitude] explode all trees

#51 altitudes

#52 #50 or #51

#53 plateau

#54 country-level

#55 #52 or #53 or #54

#56 #49 and #55

**Search strategy in Web of Science**

#01 Theme: (altitude*) OR Theme: (plateau) OR Theme: (country-level)

#02 Theme:  (Pulmonary Disease, Chronic Obstructive) OR Theme: (COPD) OR Theme: (Chronic Obstructive Pulmonary Disease) OR Theme: (COAD) OR Theme: (Chronic Obstructive Airway Disease) OR Theme: (Chronic Obstructive Lung Disease) OR Theme: (Airflow Obstruction, Chronic) OR Theme: (Airflow Obstructions, Chronic) ORTheme: (Chronic Airflow Obstructions) OR Theme: (Chronic Airflow Obstruction)

#03 Theme: (Lung Diseases, Obstructive) OR Theme: (Lung Disease, Obstructive) OR Theme: (Obstructive Lung Disease) OR Theme: (Obstructive Lung Diseases) OR Theme: (Obstructive Pulmonary Diseases) OR Theme: (Obstructive Pulmonary Disease) OR Theme: (Pulmonary Disease, Obstructive) OR Theme: (Pulmonary Diseases, Obstructive)

#04 Theme: (Bronchitis, Chronic) OR Theme: (Chronic Bronchitis)

#05 (((((((((((((((((((((((Theme: (Pulmonary Emphysema) OR Theme: (emphysema, Pulmonary)) OR Theme: (Pulmonary emphysema)) OR Theme: (Emphysema, Pulmonary)) OR Theme: (Focal Emphysema)) OR Theme: (Emphysema, Focal)) OR Theme: (emphysema, Focal)) OR Theme: (Focal emphysema)) OR Theme: (Panacinar Emphysema)) OR Theme: (Emphysema, Panacinar)) OR Theme: (emphysema, Panacinar)) OR Theme: (Panacinar emphysema)) OR Theme: (panlobar Emphysema)) OR Theme: (Emphysema, panlobar)) OR Theme: (emphysema, panlobar)) OR Theme: (panlobar emphysema)) OR Theme: (centroacinar Emphysema)) OR Theme: (centroacinar emphysema)) OR Theme: (Emphysema, centroacinar)) OR Theme: (emphysema, centroacinar)) OR Theme: (Centrilobular Emphysema)) OR Theme: (Centrilobular emphysema)) OR Theme: (Emphysema, Centrilobular)) OR Theme: (emphysema, Centrilobular))

#06 #5 OR #4 OR #3 OR #2

#07 #6 AND #1

**Search strategy in SCOPUS**

( ( TITLE-ABS-KEY ( altitude ) OR TITLE-ABS-KEY ( altitudes ) OR TITLE-ABS-KEY ( plateau ) OR TITLE-ABS-KEY ( country-level ) ) ) AND ( ( TITLE-ABS-KEY ( pulmonary AND disease, AND chronic AND obstructive ) OR TITLE-ABS-KEY ( copd ) OR TITLE-ABS-KEY ( chronic AND obstructive AND pulmonary AND disease ) OR TITLE-ABS-KEY ( coad ) OR TITLE-ABS-KEY ( chronic AND obstructive AND airway AND disease ) OR TITLE-ABS-KEY ( chronic AND obstructive AND lung AND disease ) OR TITLE-ABS-KEY ( airflow AND obstruction, AND chronic ) OR TITLE-ABS-KEY ( airflow AND obstructions, AND chronic ) OR TITLE-ABS-KEY ( chronic AND airflow AND obstructions ) OR TITLE-ABS-KEY ( chronic AND airflow AND obstruction ) OR TITLE-ABS-KEY ( lung AND diseases, AND obstructive ) OR TITLE-ABS-KEY ( lung AND disease, AND obstructive ) OR TITLE-ABS-KEY ( obstructive AND lung AND disease ) OR TITLE-ABS-KEY ( obstructive AND lung AND diseases ) OR TITLE-ABS-KEY ( obstructive AND pulmonary AND diseases ) OR TITLE-ABS-KEY ( obstructive AND pulmonary AND disease ) OR TITLE-ABS-KEY ( pulmonary AND disease, AND obstructive ) OR TITLE-ABS-KEY ( pulmonary AND diseases, AND obstructive ) OR TITLE-ABS-KEY ( bronchitis, AND chronic ) OR TITLE-ABS-KEY ( chronic AND bronchitis ) OR TITLE-ABS-KEY ( pulmonary AND emphysema ) OR TITLE-ABS-KEY ( emphysemas, AND pulmonary ) OR TITLE-ABS-KEY ( pulmonary AND emphysemas ) OR TITLE-ABS-KEY ( emphysema, AND pulmonary ) OR TITLE-ABS-KEY ( focal AND emphysema ) OR TITLE-ABS-KEY ( emphysema, AND focal ) OR TITLE-ABS-KEY ( emphysemas, AND focal ) OR TITLE-ABS-KEY ( focal AND emphysemas ) OR TITLE-ABS-KEY ( panacinar AND emphysema ) OR TITLE-ABS-KEY ( emphysema, AND panacinar ) OR TITLE-ABS-KEY ( emphysemas, AND panacinar ) OR TITLE-ABS-KEY ( panacinar AND emphysemas ) OR TITLE-ABS-KEY ( panlobular AND emphysema ) OR TITLE-ABS-KEY ( emphysema, AND panlobular ) OR TITLE-ABS-KEY ( emphysemas, AND panlobular ) OR TITLE-ABS-KEY ( panlobular AND emphysemas ) OR TITLE-ABS-KEY ( centriacinar AND emphysema ) OR TITLE-ABS-KEY ( centriacinar AND emphysemas ) OR TITLE-ABS-KEY ( emphysema, AND centriacinar ) OR TITLE-ABS-KEY ( emphysemas, AND centriacinar )

**Search strategy in OVID**

#01 (Pulmonary Disease, Chronic Obstructive or COPD or Chronic Obstructive Pulmonary Disease or COAD or Chronic Obstructive Airway Disease or Chronic Obstructive Lung Disease or Airflow Obstruction, Chronic or Airflow Obstructions, Chronic or Chronic Airflow Obstructions or Chronic Airflow Obstruction or Lung Diseases, Obstructive or Lung Disease, Obstructive or Obstructive Lung Disease or Obstructive Lung Diseases or Obstructive Pulmonary Diseases or Obstructive Pulmonary Disease or Pulmonary Disease, Obstructive or Pulmonary Diseases, Obstructive or Bronchitis, Chronic or Chronic Bronchitis or Pulmonary Emphysema or Emphysemas, Pulmonary or Pulmonary Emphysemas or Emphysema, Pulmonary or Focal Emphysema or Emphysema, Focal or Emphysemas, Focal or Focal Emphysemas or Panacinar Emphysema or Emphysema, Panacinar or Emphysemas, Panacinar or Panacinar Emphysemas or Panlobular Emphysema or Emphysema, Panlobular or Emphysemas, Panlobular or Panlobular Emphysemas or Centriacinar Emphysema or Centriacinar Emphysemas or Emphysema, Centriacinar or Emphysemas, Centriacinar or Centrilobular Emphysema or Centrilobular Emphysemas or Emphysema, Centrilobular or Emphysemas, Centrilobular).ab.

#02 (Altitude or altitudes or plateau or country-level).ab.

#03 #01 AND #02

**Search Strategy in CBM**

#01 ("高原"[常用字段] OR ("高海拔"[常用字段] OR "高海拔"[主题词]))

#02 ("慢阻肺"[常用字段] OR ("慢性阻塞性肺疾病"[常用字段] OR "慢性气道阻塞"[常用字段] OR "慢性气道阻塞性疾病"[常用字段] OR "慢性阻塞肺疾病"[常用字段] OR "COAD"[常用字段] OR "COPD"[常用字段] OR "肺疾病, 慢性阻塞性"[主题词]) OR ("COPD"[常用字段] OR "慢性阻塞性肺疾病"[常用字段] OR "慢性气道阻塞"[常用字段] OR "慢性气道阻塞性疾病"[常用字段] OR "慢性阻塞肺疾病"[常用字段] OR "COAD"[常用字段] OR "肺疾病, 慢性阻塞性"[主题词]))

#03 #01 AND #02
