# Supplementary material for: Prevalence of chronic obstructive pulmonary disease at high altitude: a systematic review and meta-analysis
Source: PeerJ. 2020 Apr 3;8:e8586. doi: 10.7717/peerj.8586 (PMC7134014; doi:10.7717/peerj.8586)
Supplement: Supplemental Information 3 [file peerj-08-8586-s003.docx]

**The Rationales**

COPD is a severe disease that could make heavy financial and social burden, and it is the third leading causing of mortality. Recently, several studies have investigated the prevalence of COPD at high altitude (>1500m). However, much remains to be understood about the correlation between altitude and COPD. Several studies reported a higher prevalence of COPD at high altitude, while inconsistent with some other studies. So conducting a meta-analysis to evaluate the prevalence of COPD at high altitude and assess whether altitude was an independent risk factor of COPD is necessary.
