# Supplementary material for: Prevalence of chronic obstructive pulmonary disease at high altitude: a systematic review and meta-analysis
Source: PeerJ. 2020 Apr 3;8:e8586. doi: 10.7717/peerj.8586 (PMC7134014; doi:10.7717/peerj.8586)
Supplement: Supplemental Information 4 [file peerj-08-8586-s004.docx]

**The Contribution**

The contribution for this meta-analysis are as follows: First, understanding the prevalence of COPD at high-altitude could offer an evidence for further studies to explore more information for COPD and we found the prevalence of COPD was higher than average data at high altitude. Moreover, making clear whether altitude is a risk factor for the prevalence of COPD could help us to take early intervention measures for the disease, delay the progress of the disease and improve the quality of life of patients.
