# Supplementary material for: Prevalence of chronic obstructive pulmonary disease at high altitude: a systematic review and meta-analysis
Source: PeerJ. 2020 Apr 3;8:e8586. doi: 10.7717/peerj.8586 (PMC7134014; doi:10.7717/peerj.8586)
Supplement: Supplemental Information 5 [file peerj-08-8586-s005.doc]

**SUPPLEMENTARY INFORMATION**

**TITLE:** Prevalence of chronic obstructive pulmonary disease at high altitude: a systematic review and meta-analysis

**Authors’ full names:** Huaiyu Xiong

**Authors’ affiliation(s):** LanZhou Univesity


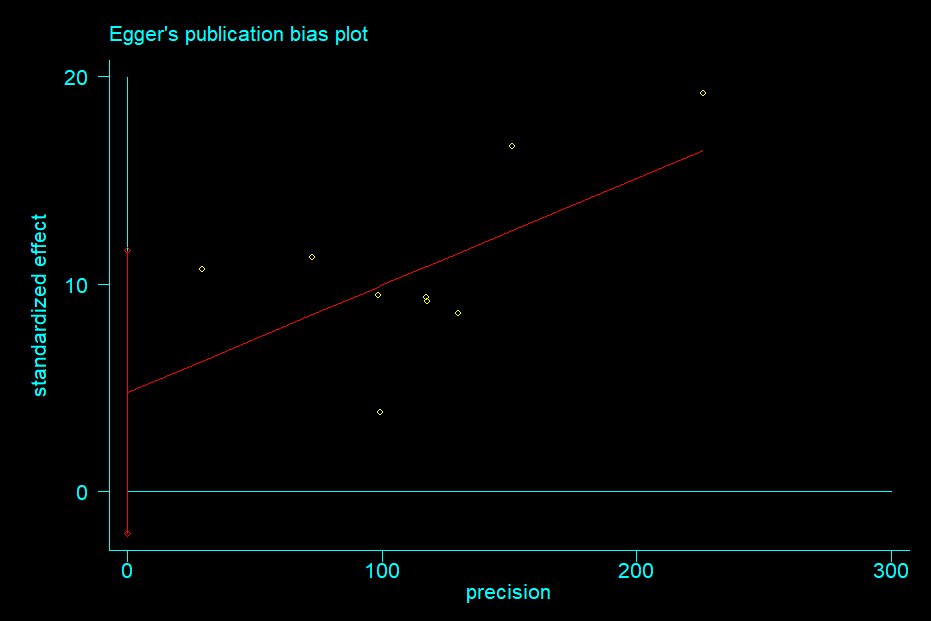


**Figure S1-** Plot for publication bias (Egger’s Test).


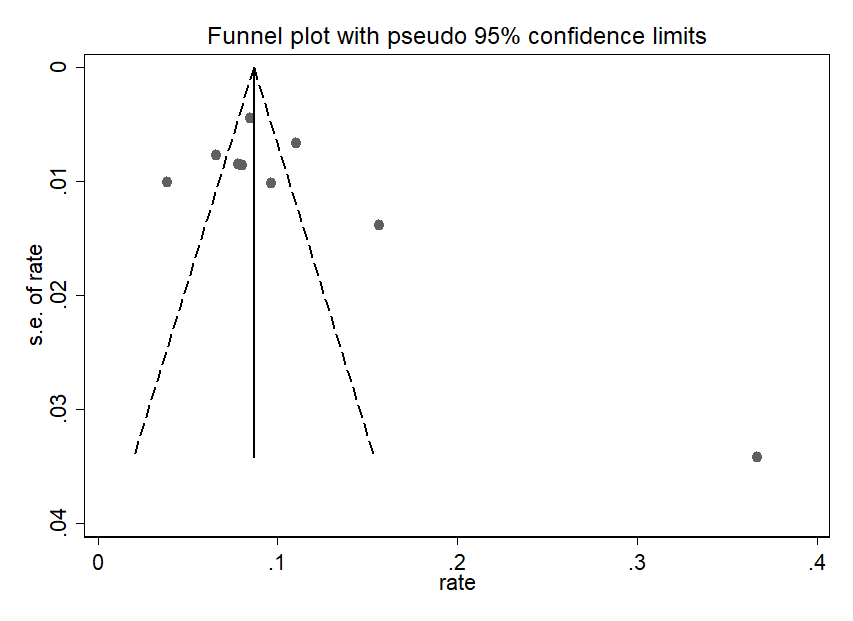


**Figure S2-** Funnel plot for the prevalence of COPD.


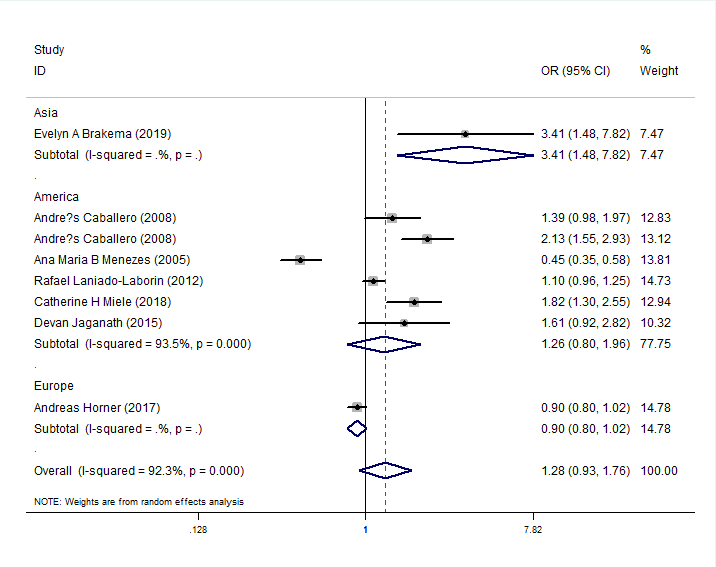


**Figure S3***-* Forest plot for detecting whether altitude was a risk factor of COPD by regions.
